# Comparison of Vertebrate *Cytochrome b* and Prepronociceptin for Blood Meal Analyses in *Culicoides*

**DOI:** 10.3389/fvets.2015.00015

**Published:** 2015-05-27

**Authors:** Leila Hadj-Henni, Thibaut De Meulemeester, Jérôme Depaquit, Philippe Noël, Adeline Germain, Remi Helder, Denis Augot

**Affiliations:** ^1^Université de Reims Champagne-Ardenne, ANSES, SFR Cap Santé, EA4688 – USC «Transmission Vectorielle et Épidémiosurveillance de Maladies Parasitaires (VECPAR)», Reims, France; ^2^Naturalis Biodiversity Center, Leiden, Netherlands; ^3^Laboratoire IAE, Université de Reims Champagne-Ardenne, Station URCA-CERFE, Boult-aux-Bois, France

**Keywords:** *Culicoides*, blood meal, PCR, *PNOC*, *Cyt b*, France

## Abstract

To date, studies on host preferences and blood meal identification have been conducted for *Culicoides* species using molecular-based methods such as PCR techniques to amplify only a fragment from universal vertebrate mitochondrial genes such as *cytochrome c oxidase subunit I* or *cytochrome b* (*Cyt b*). The vertebrate prepronociceptin gene (*PNOC*) was also tested in this field. However, the choice of molecular marker to identify blood meal is critical. The objective of our study is to compare the ability of *Cyt b* and *PNOC* as molecular markers for blood meal identification depending on the stage of blood meal digestion. In order to determine whether these *Cyt b* and *PNOC* could provide a positive result, 565 blood-fed females of *Culicoides* spp were collected and morphologically identified. The samples were collected between 2012 and 2014, in two localities in France. The collection localities were near either livestock or a forest. To catch the specimens, we used UV CDC miniature light traps. *PNOC* sequence of donkeys (*Equus asinus*) was sequenced and submitted because it was missing in GenBank. Our findings emphasize that the *PNOC* marker is not suitable to separate closely related Equid species such as horses and donkeys. The *Cyt b* marker was able to identify 204 more samples when compared to *PNOC* (99.55% of specimens). *Cyt b* appears to be better able to detect the origin of blood meals from females with digested blood in their abdomens. We conclude that *Cyt b* is a good marker as it increases the accuracy of blood meal identification of engorged females containing digested blood in their abdomens. The host opportunist behavior of *Culicoides*, especially that of *C. obsoletus* and *C. scoticus*, the main vectors of BTV in Europe was also highlighted.

## Introduction

The study of host preferences of the native population of hematophagous *Culicoides* spp is critical in assessing and taking action to prevent infectious disease outbreaks. A better knowledge of this field could help identify the animal reservoirs for arboviruses. In Europe, before BTV-8 experience, bluetongue was considered an exotic disease that could be controlled by animal movement restriction and vaccination (1). Since the spread of BTV-8 in northern Europe, despite the absence of *C. imicola*, the main African vector of BTV, some abundant autochthonous *Culicoides* species have been suspected and incriminated, mainly species from Obsoletus and Pulicaris groups (2). More recently, in autumn 2011, an unidentified disease of livestock was reported in Germany and, the etiologic agent of this disease was identified as a novel *orthobunyavirus* and named Schmallenberg virus (SBV) (3). Midges of the group Obsoletus have been shown to be natural vectors for this virus (4).

To date, many studies on host preferences have been conducted for *Culicoides* species using serological assays (5–7). However, these techniques are time consuming and have limited sensitivity and specificity (8). Molecular-based methods were also used. PCR techniques were used to amplify only a fragment from the universal vertebrate mitochondrial genes *cytochrome c oxidase subunit I* (COI) or *cytochrome b* (*Cyt b*) (9–13). A species-specific multiplex PCR assay was also developed (14, 15). The vertebrate prepronociceptin gene (*PNOC*) was also tested (16, 17). However, the proportion of successful identification of blood meal origins is variable and may be affected by the quality of the samples and the laboratory protocols.

To date, there is no study to compare these molecular markers, except that of Slama et al. (17) during which the authors showed that in the *PNOC-*PCR sequencing methods, the sensitivity of host DNA detection was lower than found by *Cyt b* PCR method. Here, to compare and to test the ability to identify the origin of blood meals taken from vertebrate hosts of both molecular markers, i.e., *PNOC* and *Cyt b*, we carried out our study on 565 wild-caught females of *Culicoides* spp trapped near livestock and forest in two localities in France. Determination of the blood meal origin was carried out through simple sequencing and Blast of the sequences in GenBank. We also compared the ability of each marker to provide interpreted sequences depending on blood digestion stage. The capacity of each marker to amplify bird DNA and to separate related animal species was also checked.

## Materials and Methods

### Collection and morphological identification of biting midges

In this study, 565 engorged *Culicoides* females were collected using UV CDC miniature light traps (John W. Hock Company, FL, USA). These traps were operated monthly in the period from 2012 to 2014, from sunset to sunrise. Three traps were positioned at a farm containing livestock (horses, sheep, cattle, and other domestic animals) as described by Lassen et al. (10) and Ninio et al. (16). The fourth trap was set in a forest area (Table 1).

**Table 1:** **Sample of blood-fed *Culicoides* in this study**.

Location	Trap	Site	Coordinates	Predominant animal species in the vicinity of the trap (*n* = effective when the counting is possible)	Number of blood-fed female
Boult-aux-Bois (Ardenne)	1	Forest	49°25′ 55″ N, 4°50′ 35″ E	Wild boar, roe deer, red deer, horses around (*n* = 10) (nearest horses were located at around 450 m from the trap)	91
Louvois (Marne)	2	Pasture	49°6′ 6″ N, 4°7′ 0″ E	Dogs (*n* = 2), cats (*n* = 2), chickens (*n* = 6), geese (*n* = 2), ducks (*n* = 4), pigs (*n* = 5), rabbits (*n* = 21), cattle (*n* = 2), goats (*n* = 2), horses (*n* = 6)	368
	3	Sheepfolds		Sheep (*n* = 4)	55
	4	Donkey’s shelter		Donkeys (*n* = 4), ponies (*n* = 2)	51

All blood-fed females of *Culicoides* were preserved in a 95% ethanol solution until their processing. To avoid cross-contamination, every dissection was performed with single-use sterile equipment. Head, genitalia, and wings were mounted between slide and cover slip to identify the specimens morphologically according to wing pattern (18). The insect identification was conducted twice by two different persons. The species as *C. obsoletus* and *C. scoticus* are difficult to distinguish morphologically and were therefore identified according to specific morphological and morphometrical characters (19). In addition, their identification was confirmed by molecular sequencing of the mitochondrial COI gene using the primers C1-J-1718 and C1-N-2191 (20) (Table 2).

**Table 2:** **Primer sequences used in the molecular analyses of blood meals and *Culicoides* species identification**.

Primers	5′–3′sequences	PCR conditions (this study)	Primer references
**Blood meal primers**
PNOC (R)	TGCCTCATAAACTCACTGAACC	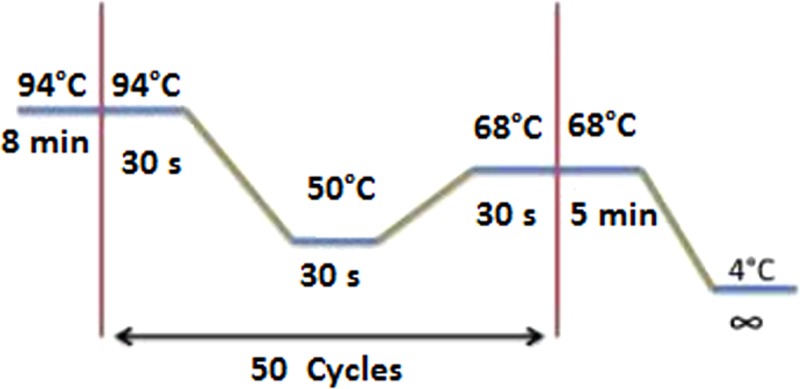	Haouas et al. (21)
PNOC (F)	GCATCCTTGAGTGTGAAGAGAA	

Cytbvert1D (R)	CCATCCAACATYTCADCATGA	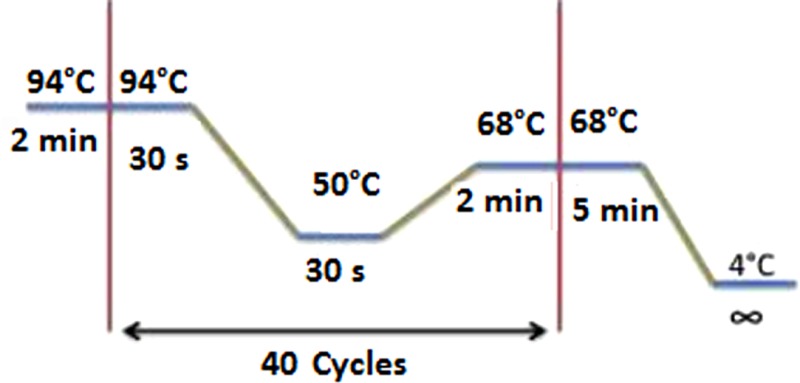	This study, modified from Kocher et al. (22)
Cytbvert2D (F)	GCHCCTCAGAATGATATTTGK	

***Culicoides* primers**
C1-N-2191 (R)	CAGGTAAAATTAAAATATAAACTTCTGG	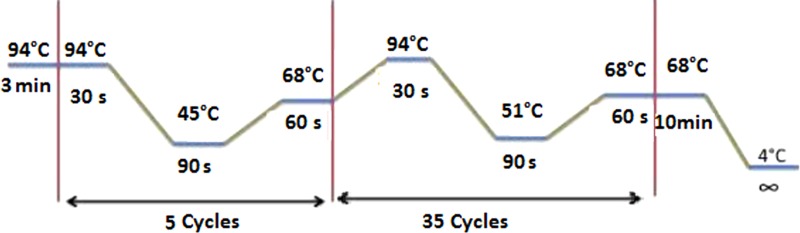	Simon et al. (20)
C1-J-1718 (F)	GGAGGATTTGGAAATTGATTAGT	

### Extraction of DNA for blood meal analyses

**Figure 1:**
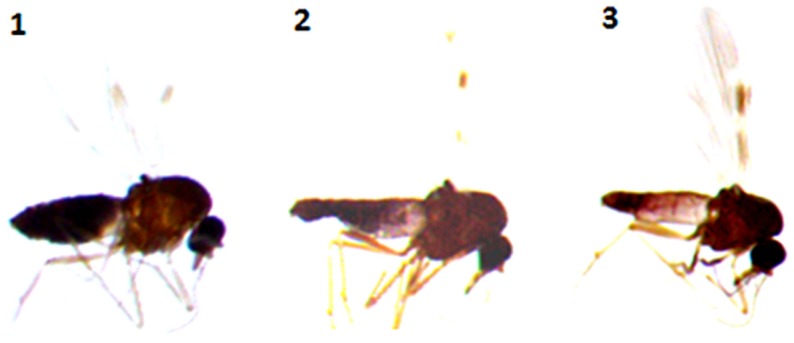
***Culicoides* females with different stages of blood meal digestion**. (1) Fully engorged females; (2) abdomen showing advanced signs of digestion; (3) abdomen showing any remainder of blood in their abdomen.

Using a conservative approach, we included all available females showing any remainder of blood in their abdomen in order to detect the maximum number of potential hosts. The color of the abdomen was observed under a magnifying glass and, if necessary, under a microscope, in order to evaluate the stage of digestion of the blood meal a visual estimation of the digestion status of *Culicoides* blood meals). We thus included: (i) fully engorged females (*n* = 220) with red, brownish-red, and dark-red abdomens (the color of the blood meal could depend on the time that passed before the midges were transferred in ethanol, and/or the length of storage in ethanol before dissection); (ii) abdomens showing advanced signs of digestion (*n* = 264); and finally (iii) females showing any remainder of blood in their abdomen (*n* = 81) (Figure 1).

Abdomens were transferred in individual, sterilized, 1.5 ml vials, and stored at −20°C. After an initial manual crushing step, the extraction was carried out using the QIamp DNA mini Kit (Qiagen, GmbH, Hilden, Germany) following the manufacturer’s instructions. The DNA was eluted in a final volume of 120 μl of AE buffer.

### Blood meal molecular analyses

The blood meal analyses were performed by sequencing the vertebrate’s *PNOC* using the primers *PNOC* (F) and *PNOC* (R) following the PCR conditions described by Haouas et al. (21). We have also used the degenerate and universal vertebrate primers from the *Cyt b* gene modified by Kocher et al. (22). The amplification conditions and the primer sequences are illustrated in the Table 2. PCRs for both markers were performed in a 50 μl volume using 10 μl of extracted DNA solution, 50 pmol of the primers, and Taq polymerase (5′, Germany).

Contamination was checked via the inclusion of a negative control in each run. Moreover, two positive controls were also processed in each run: DNA extracts from cattle (*Bos taurus*) and from Mallard ducks (*Anas platyrhynchos*). Mallard DNA was used to check the capacity of each marker to amplify bird DNA. The PCR products were examined using electrophoresis on a 2% agarose gel with ethidium bromide staining. PCR products were sequenced bi-directionally using the primers typically used for PCR.

### Sequencing and data analyses

Sequences of at least 200 bp for *PNOC* and 300 bp for *Cyt b* were compared to homologous sequences deposited in GenBank using BLAST. Blood meals were considered as identified to the species level if their sequence showed ≥98% homology with a sequence deposited into GenBank. Sequence editing and analyses were performed using BioEdit Sequence Alignment Editor V7.2.3 software.

### Statistical analyses

To test the efficiency of each marker to provide a positive result and to check if the two markers react in the same way at different stages of digestion (*PNOC* vs. *Cytb*), a chi-square analyses were carried out.

## Results

### *Culicoides* species identification

A total of 59,704 *Culicoides* biting midges [females (nulliparous, blood-fed, gravid, parous) and males] were collected by the four UV CDC miniature light traps used in this study during the period 2012–2014. The total sample included the following groups: 33,470 were nulliparous (56.05%), 565 were blood-fed (0.95%), 6173 were gravid (10.34%), 17,988 were parous (30.13%), and 1508 were male (2.53%).

In the present study, the most abundantly sampled species was *C. obsoletus* (34.64%), followed by *C. achrayi* (24.54%), *C. scoticus* (14.43%), and *C. punctatus* (13.2%). Although *C. lupicaris* (2.06%)*, C. newsteadi* (1.44%), *C. furcillatus* (5.77%), *C. brunnicans* (0.44%), *C. pulicaris* (1.44%)*, C. subfasciipennis* (1.88%)*, C. vexans* (0.2%), and *C. chiopterus* (0.4%) were less numerous in our sampling (Table 3).

**Table 3:** **Origin of blood meals from blood-fed *Culicoides* spp**.

*Culicoides* species^a^ (number by red abdomen/digested blood/any remainder of blood^b^)	Wing patterns	PNOC		Cytb	
		Red abdomens (*n* = 220)	Advanced sign of digestion (*n* = 264)	Red abdomens (*n* = 220)	Advanced sign of digestion (*n* = 264)
*C. obsoletus* (83/85/20)	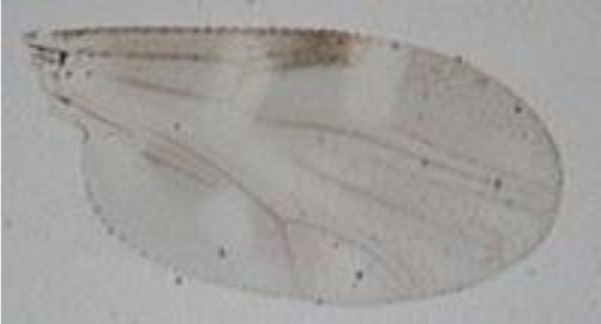	*Equus caballus* (*n* = 52)	*Bos taurus* (*n* = 1)	*Equus caballus* (*n* = 80)	*Equus caballus* (*n* = 23) *Bos taurus* (*n* = 3)
		*Homo sapiens* (*n* = 2)	*Homo sapiens* (*n* = 2)	*Equus asinus* (*n* = 1)	*Homo sapiens* (*n* = 38)
			*Equus caballus* (*n* = 1)	*Homo sapiens* (*n* = 2)	*Felis silvestris* (*n* = 1)

*C. scoticus* (27/43/25)	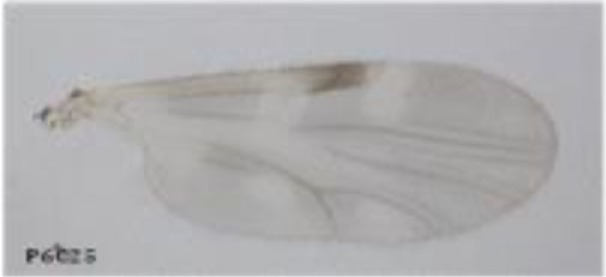	*Equus caballus* (*n* = 23)	–	*Equus caballus* (*n* = 26)	*Equus caballus* (*n* = 1)
		*Homo sapiens* (*n* = 1)		*Homo sapiens* (*n* = 1)	*Homo sapiens* (*n* = 2)

*C. chiopterus* (1/1/5)	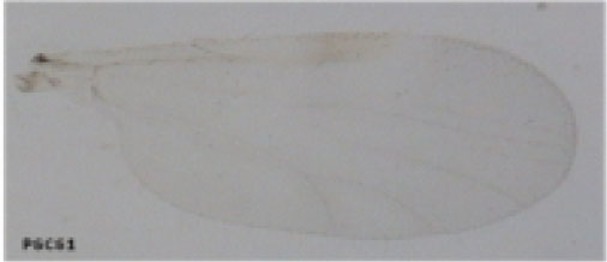	*Equus caballus* (*n* = 1)	*Bos taurus* (*n* = 1)	*Equus caballus* (*n* = 1)	*Bos taurus* (*n* = 1)

*C. lupicaris* (7/3/9)	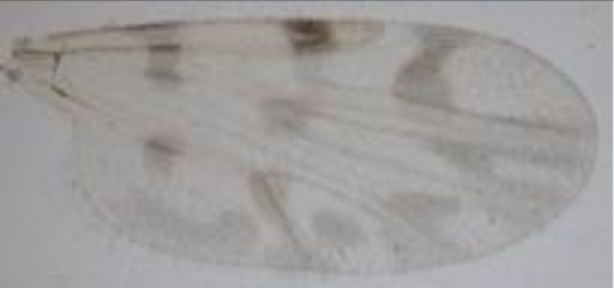	*Equus caballus* (*n* = 7)	–	*Equus caballus* (*n* = 6)	*Equus caballus* (*n *= 1)
					*Equus asinus* (*n *= 1)
				*Equus asinus* (*n* = 1)	*Homo sapiens* (*n* = 1)

*C. pulicaris* (1/6/11)	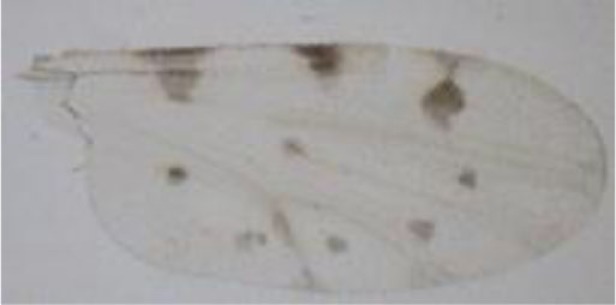	*Equus caballus* (*n* = 1)	*Bos taurus* (*n* = 1)	*Equus caballus* (*n* = 1)	*Equus caballus* (*n* = 3)
					*Bos taurus* (*n* = 1)
					*Homo sapiens* (*n* = 2)

*C. newstead (2//5/0)*	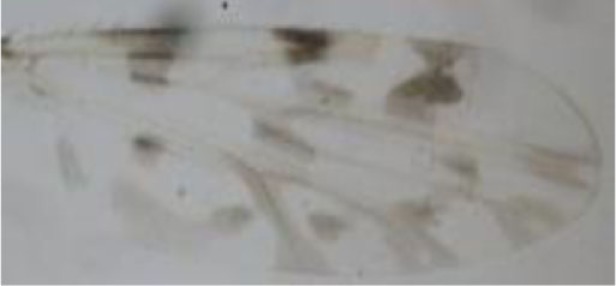	*Equus caballus* (*n* = 2)	–	*Equus caballus* (*n* = 2)	*Equus caballus* (*n* = 2)
					*Equus asinus* (*n* = 1)
					*Homo sapiens* (*n* = 1)

*C. punctatus* (26/38/1)	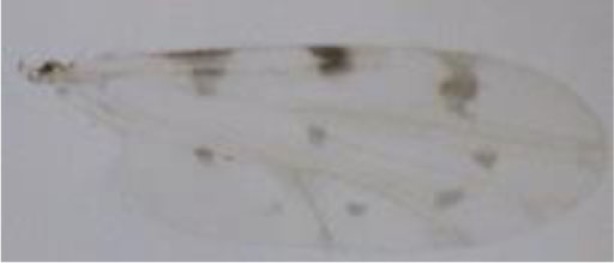	***Equus caballus*** **(*n* = 25)**	*Equus caballus* (*n* = 1)	*Equus caballus* (*n* = 20)	*Equus caballus* (*n* = 13)
		*Homo sapiens* (*n* = 1)		***Equus asinus*** (***n**** = 5)*	***Equus asinus*** (***n**** = 8)*
				*Homo sapiens* (*n* = 1)	*Homo sapiens* (*n* = 4)

*C. achray* (63/55/7)	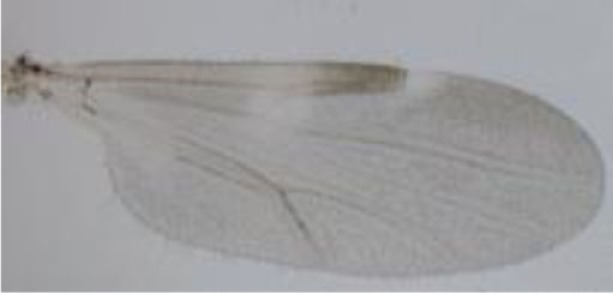	*Equus caballus* (*n* = 62)	–	*Equus caballus* (*n* = 61)	*Equus caballus* (*n* = 38)
				***Equus asinus*** (***n**** = 1)*	*Equus asinus* (*n* = 2)
				***Gallus gallus*** (***n**** = 1)*	*Homo sapiens* (*n* = 9)

*C. furcillatus* (5/23/3)	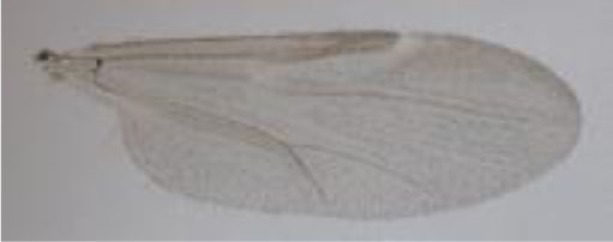	*Equus caballus* (*n* = 5)	–	*Equus caballus* (*n* = 5)	*Equus caballus* (*n* = 8)
					*Homo sapiens* (*n* = 8)

*C. subfasciipennis* (4/3/0)	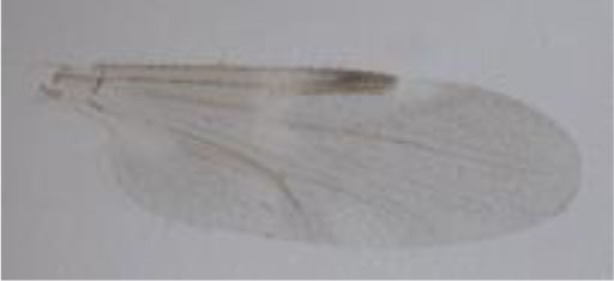	*Equus caballus* (*n* = 2)	–	*Equus caballus* (*n* = 4)	*Homo sapiens* (*n* = 3)

*C. brunnicans* (1/1/0)	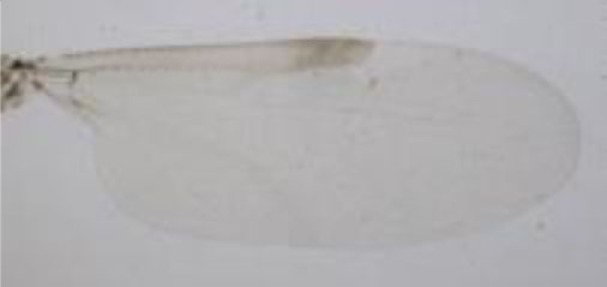	–	–	–	*Equus caballus* (*n* = 1)

*C. vexans* (0/1/0)	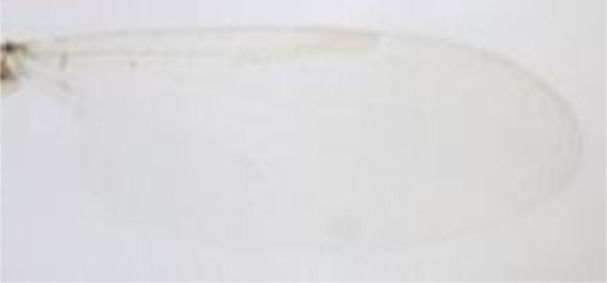	–	–	–	*Homo sapiens* (*n* = 1)

Total		184	7	219	176

*^a^The same data set was tested for both markers*.

*^b^Sample with any remainder of blood (*n* = 81) are not represented in the table because they provide all a negative or not interpretable results with both markers*.

### Blood meals analyses

From the 565 *Culicoides* identified as blood-fed, at least 11 different species were found to have fed on horses (73.92% for *Cyt b* and 95.28% for *PNOC*), 5 different species on donkeys (*Equus asinus*), 4 different species on cattle, only 1 on a cat (*Felis silvestris*), and 1 on a chicken (*Gallus gallus*). Finally, *Cyt b* analyses show that 19.20% of *Culicoides* fed on humans (*Homo sapiens*) vs. 4.81%, the result established with *PNOC* (Table 3).

**Figure 2:**
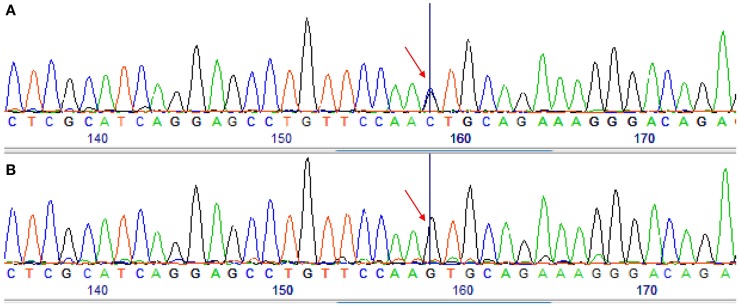
**Sequencing chromatograms obtained by sequencing of the *PNOC*-PCR products for two different samples**. **(A)** A sample identified as *Equus asinus* by *Cytb* sequencing. **(B)** A sample identified as *Equus caballus* by *PNOC* and *Cytb* sequencing. Arrow shows the difference between the two samples.

Analyses from *Cyt b* show that all *C. punctatus*, which were caught in Boult-aux-Bois (forest) (*n* = 13) (Table 3), are the main species that took blood meals from donkeys. Further, we observed in GenBank two haplotypes of horse sequences with a *PNOC* gene differing by a single base (e.g., sequences under the accession numbers XM_001493120 and AY011855) (Figure 2). We noted that the sequence of donkeys was missing. Consequently, we provided it from donkey blood following the protocol previously described (GenBank accession number KM521860). We compared the donkey blood and found it to be identical to the first haplotype of *Equus caballus* (accession number XM_001493120). This led us to the conclusion that the *PNOC* gene is unable to distinguish *E. caballus* sequences from those of *E. asinus* (99% homology) whereas *Cyt b* is able to distinguish both species.

Equally, no sequences were reported from *PNOC* using the Mallard DNA as opposed to the successful amplification that was reported using *Cyt b*. Evidence of mixed blood meals was not observed in the present study.

In our study, analyses from *PNOC* and *Cyt b* genes detected blood meals of all 220 fully engorged females, 184 (83.63%) and 219 (99.55%), respectively. During the analyses from females showing advanced signs of blood digestion (*n* = 264), *PNOC* provided only seven sequences (2.65%) whereas *Cyt b* identified 176 blood meals (66.67%). No identification was reported from either marker on abdomens containing only traces of blood.

Statistical analyses from *Cytb* (χ^2^ = 281.3717, df = 2, *p* < 2.2e−16) and *PNOC* (χ^2^ = 399.9959, df = 2, *p* < 2.2e−16) show a very low *p*-value for both markers, meaning that the success of the two markers to provide a positive result is different depending the stage of digestion. Comparison of the difference in success between the two markers (*PNOC* vs. *Cytb*) provided a zero value of *p*-value (χ^2^ = 1717.429, df = 2, *p* = 0) meaning that markers do not react the same way facing the stage of digestion.

## Discussion

In the present study, the PCR technique based on the *Cyt b* gene of mitochondrial DNA (mtDNA) and the *PNOC* was selected for blood meal identification: (i) fully engorged *Culicoides*, (ii) *Culicoides* showing an advanced sign of digestion, and (iii) females showing any remainder of blood in their abdomen. The objective was to determine the ability of each marker to detect DNA from vertebrate hosts in the abdomens of *Culicoides* depending on the stage of blood meal digestion. Our results show that the *Cyt b* marker was more efficient (66%) than *PNOC* to detect the origin of blood meals in advanced stages of digestion; this could be explained by the fact that immature erythrocytes have lost their mitochondria during the final stages of maturation, which are found in abdomens of engorged arthropods (23). Several studies have also highlighted that there is a significant negative relationship between the time since ingestion and the success of analyses (21, 24–26). It is interesting to note that most studies in this field include only fully engorged females. The lack of identification of blood meal origin by both markers in digested blood and in abdomens with some traces of blood can be explained by the fact that host DNA was extensively degraded during digestion. Kirsten and Gray (27) in their study on engorged *Ixodes ricinus* ticks, a vector of Lyme disease, explain that nested PCR amplification of PCR product could increase detection sensitivity and the limit of detection to 40 days post-digestion (27).

In the present study, no interpretable sequences were reported with both markers, perhaps due to contamination even though the samples were prepared under sterile conditions. DNA purification prior to PCR would be necessary for these samples and the use of specific-species primers could increase the yield of successful identification (14).

More than 19% of our positive samples gave sequences that matched *Homo sapiens* in our study. In order to check contamination, it would be better to test *Culicoides* provided sequences matching *Homo sapiens* consistently with human-specific primers that amplify a segment of the hyper variable mitochondrial control region (28). In fact, it was not expected that primers amplifying specific segments of human mtDNA would also amplify the corresponding segments of mtDNA from other species (22).

In our study, no identification conflicts were reported between *PNOC* and *Cyt b* markers except blood meals originating from donkeys and horses because the *PNOC* marker failed to separate donkey and horse DNA. The same findings were reported in the study of Ninio et al. (16), where no differentiation was made with *PNOC* between wild boars (*Sus scrofa scrofa*) and pigs (*Sus scrofa*). We conclude that *PNOC* is not suitable to separate closely related species such as Equidae; this marker is not a good candidate to be used alone. We confirm also the findings of Haouas et al. (21) and Slama et al. (17), in which *PNOC* is only suitable to detect blood meal origin from mammals but not from birds. In addition, the *PNOC* gene underperforms in detecting blood meal origin, especially from abdomens with digested blood. Consequently, we confirm that in this type of study, the gene of choice is the *Cyt b*, because it is present in numerous copies in each cell (29). It is also known that vertebrate mtDNA can evolve faster than nuclear DNA, thus they are more polymorphic and more useful for species identification (22). The *Cyt b* marker has also been used successfully on ticks (27, 30), mosquitoes (31, 32), tsetse flies (33), sand-flies (34), and *Culicoides* (12, 13). In most of these studies, *Cyt b* has proven its discriminating power although in some studies, no differentiation has been made between domestic pigs and wild boars (*Sus scrofa*), sheep and mouflon (*Ovis gmelini musimon*), and ibex (*Capra ibex*) and goat (*Capra aegagrus*) because of their close phylogenetic heritage (14). RFLP analysis based on *Cyt b* sequences has been limited to discriminating animals of the same genus, namely, *C. elaphus* and *C. nippon*. However, analyses of the available sequence data indicate that this problem could be resolved, because sufficient variability exists in the *Cyt b* sequence to design specific probes, which could distinguish between related species (27). In the present study, our degenerate primers discriminated *E. caballus* and *E. asinus*, two closely related species from the same genus (*Equus*).

In the study of Pettersson et al. (12), in order to increase the opportunity for host identification, the authors added a second widely studied gene COI for blood meal analysis for samples that did not produce results with *Cyt b* primers. Consequently, identification of *Culicoides* blood meals using the *Cyt b* gene coupled with COI gene for vertebrates is a logical application since the COI and *Cyt b* DNA barcoding regions from more species are being sequenced and submitted to public databases. The capacity for identification of blood meals via both markers will become more and more precise.

In the present study, our sampling strategy was to use light traps close to livestock as suggested by Garros et al. (14) to maximize the capture of blood-fed females on the hosts present at study sites (wild fauna for Boult-aux-Bois and domestic fauna for Louvois). Although most studies on host preferences of *Culicoides* found that large mammals, specifically ruminants, were the preferred host for biting midges (*Culicoides spp*.) (9–11, 16).

However, we observed the susceptibility of horses and ponies (=*E. caballus*) to the attacks of *Culicoides* biting midge species (more than two-thirds of blood-fed *Culicoides*, examined with both markers, fed on horses). This observation could be expected given that horses and ponies were the most common hosts available in the vicinity of the four trap locations. In fact, every trap had horse as the most numerous blood meal host. Even the trap in the forest caught a higher number of specimens with blood meals originating from horses than any other potential hosts even though no horse was nearer than 450 m. In addition, *C. obsoletus* was the most common species found to have fed on horses () suggesting that this species is prone to opportunistic feeding behavior as it is known to be attracted to horses, cattle, and sheep (9, 10, 35–38). Host-feeding behavior of *Culicoides* species may also be influenced by semiochemicals factors, and even shape or size of potential hosts (39–41). In the present study, no mixed blood meals were detected. Our results also suggest that other species than the putative vectors can feed on horses, including *C. furcillatus*, *C. subfasciipennis*, *C. brunnicans*, and *C. achrayi*. There are poor studies on those species maybe because is not predominant in light traps’ catches (16). In our study, *C. achrayi* was abundant. It would also be interesting in further studies to assess the variety of hosts through diversification of trapping sites and a larger sampling. The investigation on host-feeding pattern of those species and on other species of *Culicoides* in general is important in understanding the epidemiology of *Culicoides* transmitted diseases, which affect domestic as horses and wild animals as well.

Analyses from *Cyt b* show that some blood-fed females (*C. punctatus*) that were caught on Boult-aux-Bois took blood meals from donkeys; note that a donkey was closer than ∼1.3 km from where the blood meal was collected (trap N°1, Table 1). Lassen et al. (10) collected *Culicoides* fed on cattle in a forest area 800 m from the nearest cattle; they also collected *Culicoides* fed on sheep 1.5 km from the nearest sheep. This is another example, of blood-fed biting midges traveling long distances after their blood meal. It also indicates that blood-engorged *Culicoides* species may engage in long-distance dispersal, engorged females were still able to cover a distance of about 200 m (42). This may have been caused by wind drift; indeed, it is known that midges are easily carried by the wind stream.

Unfortunately, no females fed on wild animals were found, the same observations were reported by Bartsch et al. (42), Garros et al. (14), and Ninio et al. (16). It can be assumed that biting midges prefer domestic livestock if available. In addition, as most species of *Culicoides* are displaying crepuscular or nocturnal behavior (43), they may prefer to bite domestic animals resting at night instead of wild animals in movement.

Since the study of blood meals is also an important component in understanding the pathogenic role of hematophagous insects, especially *Culicoides*, the present study indicates that the PCR direct sequencing system using universal primer sets for vertebrate *Cyt b* gene is a promising technique for blood meal identification especially because: (i) *Cyt b* mtDNA has a multicopy loci (8000 copy per cell) (44) and (ii) primers used for the amplification of the *PNOC* gene do not recognize birds and reptiles DNA (17, 45). It is more judicious then to concentrate efforts to provide a single homogeneous protocol to render studies comparable. Supplementary studies on the diurnal activity of *Culicoides* and their breeding sites and capturing methods could contribute to the understanding of their feeding behavior and provide additional information on their role in the transmission of pathogens.

## Author Contributions

LH-H, JD, TM, and DA designed this study, carried out data collection, data analysis, data interpretation, and composed the paper. PN, RH, and AG carried out data collection and data analysis.

## Conflict of Interest Statement

The authors declare that the research was conducted in the absence of any commercial or financial relationships that could be construed as a potential conflict of interest.
